# Nasal mucosal microRNA expression in children with respiratory syncytial virus infection

**DOI:** 10.1186/s12879-015-0878-z

**Published:** 2015-03-25

**Authors:** Christopher S Inchley, Tonje Sonerud, Hans O Fjærli, Britt Nakstad

**Affiliations:** Department of Pediatric and Adolescent Medicine, Akershus University Hospital, 1478 Lørenskog, Norway; Institute of Clinical Medicine, University of Oslo, 0316 Oslo, Norway; Department of Clinical Molecular Biology and Laboratory Sciences (EpiGen), Akershus University Hospital, 1478 Lørenskog, Norway

**Keywords:** Respiratory syncytial virus, MicroRNA, miR-34, miR-125, miR-429, miR-155, Bronchiolitis, Children, Pediatric, Nasal mucosa

## Abstract

**Background:**

Respiratory syncytial virus (RSV) infection is a common cause of pediatric hospitalization. microRNA, key regulators of the immune system, have not previously been investigated in respiratory specimens during viral infection. We investigated microRNA expression in the nasal mucosa of 42 RSV-positive infants, also comparing microRNA expression between disease severity subgroups.

**Methods:**

Nasal mucosa cytology specimens were collected from RSV-positive infants and healthy controls. 32 microRNA were selected by microarray for qPCR verification in 19 control, 16 mild, 7 moderate and 19 severe disease samples.

**Results:**

Compared to healthy controls, RSV-positive infants downregulated miR-34b, miR-34c, miR-125b, miR-29c, mir125a, miR-429 and miR-27b and upregulated miR-155, miR-31, miR-203a, miR-16 and let-7d. On disease subgroups analysis, miR-125a and miR-429 were downregulated in mild disease (p = 0.03 and 0.02, respectively), but not in severe disease (p = 0.3 and 0.3).

**Conclusion:**

microRNA expression in nasal epithelium cytology brushings of RSV-positive infants shows a distinct profile of immune-associated miRNA. miR-125a has important functions within NF-κB signaling and macrophage function. The lack of downregulation of miR-125a and miR-429 in severe disease may help explain differences in disease manifestations on infection with RSV.

## Background

Respiratory syncytial virus (RSV) infection occurs in the majority of children during infancy [1]. Most children have mild upper respiratory symptoms such as coryza and cough. However, 2–3% of infants with RSV infection are admitted to hospital, presenting clinically with bronchiolitis or pneumonia [2]. Efforts to develop an effective vaccine have led to extensive investigation of the immune response to RSV. Patient-based immunological investigations in RSV disease commonly use the nasal mucosa as a proxy for the lower respiratory tract since the lower airways are rarely available for research purposes, and because limiting investigations only to intensive care patients may result in selection bias. RSV preferentially infects epithelial cells of the respiratory mucosa, initiating an innate immune reaction with infiltration of granulocytes and other myeloid cells, production of a range of cytokines and activation of dendritic cells [3].

microRNAs (miRNAs) are non-coding small RNA sequences that inhibit translation of messenger RNA (mRNA) to protein by binding to specific target mRNA [4]. Studies in recent years have demonstrated their role in the regulation of multiple biological pathways within the innate and adaptive immune systems. These pathways include negative feedback mechanisms to prevent an excessive immune reaction, pathways governing cell fate decisions and communication between innate and adaptive immune systems [5,6]. Several studies have investigated miRNA responses to RSV in-vitro [7-9], but to date there are no published investigations of miRNA regulation in clinical RSV disease. We have previously described an association between downregulation of *Dicer* at birth and severe infantile RSV disease [10]. Since Dicer is a key protein in miRNA biogenesis, we considered that differential *Dicer* expression on infection may result in altered miRNA profiles, which may in turn explain why some children develop severe disease.

The aim of this investigation was two-fold: i) to describe miRNAs involved in the immune response to RSV in a clinical setting; ii) to discover differences in miRNA expression between disease severity groups. We have therefore profiled miRNA in cytology brushings of the nasal mucosa in infants with RSV disease, comparing them to healthy infants.

## Methods

### Patient selection

Study inclusion was during the RSV season in Akershus, Norway, from January to March 2011. Infants < 12 months of age examined on the pediatric emergency unit at our hospital with an upper or lower airways infection were considered for inclusion. A nasopharyngeal aspirate (NPA) for virus detection and nasal mucosal cytology specimens were taken from eligible patients on admission. Those testing positive for RSV by either rapid antigen testing or multiplex RT-PCR were included in the study.

### Exclusion criteria

Patients born before 34 weeks gestation, with bronchopulmonary dysplasia, chronic lung disease including treated asthma, neurological disease, Down syndrome, hypotonia, failure to thrive, or other specific conditions likely to contribute to a more severe course of disease were excluded from the study.

### Respiratory distress

Patients were included prospectively and assessed by the treating pediatrician for respiratory distress using a modified Respiratory Distress Assessment Instrument (m-RDAI), developed for this study from a validated scoring system to allow a single measure of respiratory rate [11]. The m-RDAI is described further in Table 1.

**Table 1:** **Modified respiratory distress assessment instrument (m-RDAI)**

**Points**	**0**	**1**	**2**	**3**	**4**
**Wheeze**					
**Expiration**	None	End-Expiratory	1/2	3/4	Whole expiration
**Inspiration**	None	Partly	Whole inspiration		
**Location**	None	≤2 of 4 lung fields	≥3 of 4 lung fields		
**Retractions**					
**Supraclavicular**	No	Mild	Moderate	Significant	
**Intercostal**	No	Mild	Moderate	Significant	
**Subcostal**	No	Mild	Moderate	Significant	
**Respiratory Rate**		**Upper limit of normal respiratory rate**		**Points**	
**Age < 1 month**		50/ min		1 point for each increment of 5 breaths/minute above normal, max. 8 points	
**Age 1 – 5 months**		40/ min			
**Age 6 – 11 months**		30/ min			

Note: Maximum score for wheeze - 8 points; for retractions – 9 points; for respiratory rate – 8 points. Thus RR, retractions and wheeze are equally weighted. Maximum m-RDAI score is 25 points. Adapted from Lowell et al. [11]. Example - a 3-month old child with a respiratory rate of 62 receives 5 points for Respiratory rate (62 – 40 = 22; 22/5 = 4.4; round up to 5).

### Disease severity

For disease severity subgroup analyses, infants were classified into mild, moderate or severe disease groups. The severe disease group included infants requiring mechanical ventilation, CPAP, supplemental oxygen or supplemental fluids (intravenous or nasogastric tube). Infants who were admitted and who did not receive any of the treatments described for severe disease were classified with mild disease if they had an m-RDAI ≤ 8 and with moderate disease if they had an m-RDAI 9 – 25. All infants who were not admitted were classified with mild disease, irrespective of m-RDAI score.

### Control group

A control group of infants was recruited during routine visits to family health clinics in our catchment area. Control infants were included if they were healthy, without signs of upper or lower respiratory disease.

### Nasal samples for virus and miRNA analysis

NPAs were taken from both nostrils by deep nasal suctioning. RSV infection was confirmed using a rapid antigen test (BinaxNOW RSV Card, Alere, Waltham, Massachusetts, USA) and/or in-house RT-PCR. After removal of respiratory secretions by NPA, nasal epithelial cells were sampled from each nostril by rotating a cytology brush (Medscand Medical Cytobrush Plus, CooperSurgical, Trumbull, Connecticut, USA) over the anterior nasal mucosa. Brushes were immediately immersed in RNA stabilization reagent RNAlater (Catalog Number R0901, Sigma-Aldrich, Saint Louis, Missouri, USA). Epithelial cells were detached from the brushes and stored at −80°C in RNAlater for miRNA analysis.

### Cytology

Cytology specimens from 7 infants with mild, and 5 infants with severe disease were smeared onto a microscopy slide for visual assessment of cell type.

### RNA isolation

Epithelial cells stored at −80°C were pelleted before homogenization in QIAzolLysis Reagent (Qiagen, Hilden, Germany). The miRNeasy mini kit (Qiagen) was then used according to the manufacturer’s instructions with additional DNase treatment. Because of low yield in the control samples, these were pooled into age and gender-matched pairs during RNA isolation.

### RNA quality control

We used the NanoDrop ND-1000 Spectrophotometer (NanoDrop Technologies) and Agilent 2100 Bioanalyzer to assess RNA purity and integrity using the 260/280 ratio, the 260/230 ratio and the RNA integrity number (RIN).

### miRNA Microarray

miRNA expression profiling was performed on 48 samples on Agilent Human miRNA Microarray Release 14.0, 8x15K slides using Agilent’s miRNA Microarray System protocol together with miRNA Spike-In Kit and miRNA Complete Labeling and Hyb Kit. A single color array was used. Arrays were scanned on Agilent G2565BA Microarray Scanner. Data collection and quality assessment were performed using Agilent Feature Extraction Software v8.5. Microarray results are deposited in NCBI’s Gene Expression Omnibus [12] with accession number GSE62306.

### miRNA qPCR

miRNA differentially expressed between control and disease groups were selected from the microarray for qPCR verification. qPCR was done using looped miRNA-specific RT-primers and sequence-specific TaqMan miRNA Assays from Life Technologies (Table 2). The protocol for creating custom RT and pre-amplification pools using TaqMan Assays was used. RT and PreAmp were performed according to protocol using 60 ng total RNA in the RT-reaction. The PreAmp product was diluted 32,8 times and 4 μl were run in a total reaction volume of 25 μl in qPCR. The final concentration of TaqMan MicroRNA Assay used was 1х together with 1х TaqMan Universal Master Mix II, with UNG and nuclease-free water. The reverse transcription (RT) pool was made combining 10 μl of each 5хRT primer in a total volume of 1000 μl with 1хTE. 10 μl of each 20х TaqMan MicroRNA Assay were combined in a total volume of 1000 μl with 1хTE in the pre-amplification. Samples were run in duplicates on ABI PRISM 7900 HT Fast qPCR system and analyzed using ABI Prism SDS2.4 software (Life Technologies). RNU24 was selected as a reference gene for normalization after pre-validation for expression stability in a sub-set of our samples. Samples were normalized and calibrated using the ΔΔCq method.

**Table 2:** **miRBase IDs and mature miRNA sequences for miRNAs included in the qPCR verification**

**miRBase ID**	**Accession** ^1^	**Assay** ^2^	**Mature miRNA Sequence**
hsa-let-7d-5p	MI0000065	002283	AGAGGUAGUAGGUUGCAUAGUU
hsa-let-7f-5p	MIMAT0000067	000382	UGAGGUAGUAGAUUGUAUAGUU
hsa-let-7g-5p	MIMAT0000414	002282	UGAGGUAGUAGUUUGUACAGUU
hsa-let-7i-5p	MIMAT0000415	002221	UGAGGUAGUAGUUUGUGCUGUU
hsa-miR-16-5p	MIMAT0000069	000391	UAGCAGCACGUAAAUAUUGGCG
hsa-miR-19a-3p	MIMAT0000073	000395	UGUGCAAAUCUAUGCAAAACUGA
hsa-miR-21-5p	MIMAT0000076	000397	UAGCUUAUCAGACUGAUGUUGA
hsa-miR-23b-3p	MIMAT0000418	000400	AUCACAUUGCCAGGGAUUACC
hsa-miR-26b-5p	MIMAT0000083	000407	UUCAAGUAAUUCAGGAUAGGU
hsa-miR-27b-3p	MIMAT0000419	000409	UUCACAGUGGCUAAGUUCUGC
hsa-miR-29c-3p	MIMAT0000681	000587	UAGCACCAUUUGAAAUCGGUUA
hsa-miR-30b-5p	MIMAT0000420	000602	UGUAAACAUCCUACACUCAGCU
hsa-miR-30d-5p	MIMAT0000245	000420	UGUAAACAUCCCCGACUGGAAG
hsa-miR-31-5p	MIMAT0000089	002279	AGGCAAGAUGCUGGCAUAGCU
hsa-miR-34b-5p	MIMAT0000685	000427	UAGGCAGUGUCAUUAGCUGAUUG
hsa-miR-34c-5p	MIMAT0000686	000428	AGGCAGUGUAGUUAGCUGAUUGC
hsa-miR-96-5p	MIMAT0000095	000186	UUUGGCACUAGCACAUUUUUGCU
hsa-miR-125a-5p	MIMAT0000443	002198	UCCCUGAGACCCUUUAACCUGUGA
hsa-miR-125b-5p	MIMAT0000423	000449	UCCCUGAGACCCUAACUUGUGA
hsa-miR-130a-3p	MIMAT0000425	000454	CAGUGCAAUGUUAAAAGGGCAU
hsa-miR-146a-5p	MIMAT0000449	000468	UGAGAACUGAAUUCCAUGGGUU
hsa-miR-148a-3p	MIMAT0000243	000470	UCAGUGCACUACAGAACUUUGU
hsa-miR-155-5p	MIMAT0000646	002623	UUAAUGCUAAUCGUGAUAGGGGU
hsa-miR-183-5p	MIMAT0000261	002269	UAUGGCACUGGUAGAAUUCACU
hsa-miR-200b-5p	MIMAT0004571	002274	CAUCUUACUGGGCAGCAUUGGA
hsa-miR-203a	MI0000283	000507	GUGAAAUGUUUAGGACCACUAG
hsa-miR-205-5p	MIMAT0000266	000509	UCCUUCAUUCCACCGGAGUCUG
hsa-miR-223-3p	MIMAT0000280	002295	UGUCAGUUUGUCAAAUACCCCA
hsa-miR-324-3p	MIMAT0000762	002161	ACUGCCCCAGGUGCUGCUGG
hsa-miR-331-3p	MIMAT0000760	000545	GCCCCUGGGCCUAUCCUAGAA
hsa-miR-375	MI0000783	000564	UUUGUUCGUUCGGCUCGCGUGA
hsa-miR-429	MI0001641	001024	UAAUACUGUCUGGUAAAACCGU

^1^miRBase accession number [41].

^2^Life Technologies’ assay ID.

### Statistical analysis

Microarray results were analyzed using the R computing environment [13]. The AgiMiRNA package with quantile normalization [14] was used for pre-processing. Microarray performance was assessed using relative log expression and principal component analysis. The Linear models for microarray (Limma) package with an empirical Bayes approach [15] was used for statistical analysis. P-values were adjusted for multiple testing using a false discovery rate of 5%. Adjusted p-values < 0.05 were considered statistically significant.

PCR results were analyzed using IBM SPSS 20.0 software and the R computing environment. First, RSV positive samples were compared to controls using a multivariate linear regression model with adjustment for the RNA integrity number (RIN). There were no significant correlations between age or gender and individual miRNA expression or disease group. These variables were therefore not included in the multiple regression analysis. Significance values were adjusted for multiple testing using a false discovery rate of 5%.

Secondly, for each miRNA, boxplots of control and disease subgroups were created. miRNAs that showed visual evidence of differential expression between disease groups were selected for analysis using one-way ANOVA (adjusted for a false discovery rate of 5%), followed by Dunnett’s test, comparing all disease groups to the control group.

### Ethical issues

Written, informed consent was obtained from the guardians of all infants prior to inclusion. The Regional Committee for Medical and Health Research Ethics, South-East Norway and the data-protection officer at Akershus University Hospital approved the study protocol.

## Results

Nasal samples were taken from 105 eligible RSV-positive infants, including 40 with mild, 25 with moderate and 40 with severe disease. Fifty-nine control samples were available. 61 samples had sufficient yield and RNA quality for qPCR analysis.

The 12 samples assessed by microscopy had an abundance of squamous and ciliary epithelial cells, and granulocytes, confirming that our samples included nasal mucosal cells, not simply mucous secretions. There were no obvious differences between mild and severe disease.

### Microarray

**Figure 1:**
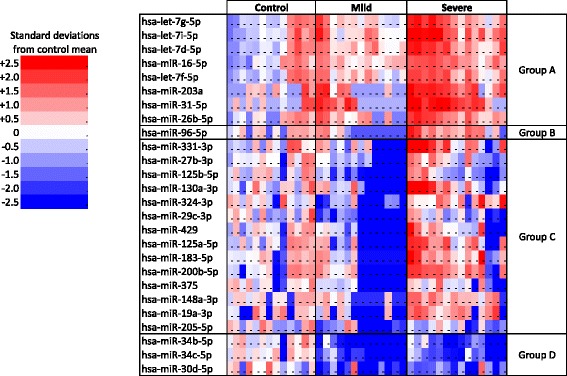
**miRNA Microarray of nasal mucosa brushings in infants infected with** ***Respiratory syncytial virus*** **, compared to healthy controls.** Note: Only miRNAs significant in the microarray (p < 0.05, adjusted for a false discovery rate of 5%) are shown. For each miRNA, results are calibrated to the mean of the control group for that miRNA. Number of samples: Control 13, Mild RSV disease 13; Severe RSV disease 14. miRNAs are grouped according to results of Limma analysis, as compared to the control group: Group A: Upregulation in RSV disease. Group B: Upregulation in severe disease; downregulation in mild disease. Group C: Downregulation in mild disease; not regulated in severe disease. Group D: Downregulation in RSV disease.

14 severe RSV, 13 mild RSV and 13 control samples were analyzed. Of 887 miRNA included on the microarray; 190 were detectable. Limma analysis of control, mild and severe disease revealed 14 differentially expressed miRNA. A further 12 miRNAs were differently expressed in pairwise comparisons between groups. Results for significantly regulated miRNA are presented in Figure 1 as a heatmap. miRNA are classified according to the pattern of expression. These 26 miRNA were selected for qPCR verification alongside miR-23b, miR-30b, miR-223, miR-146a, miR-155 and miR-21, which have well described roles in the immune system but were not differentially expressed in the microarray.

### qPCR

Fourty-two RSV-positive and 19 control samples with were analyzed by qPCR, including 16 with mild, 7 with moderate and 19 with severe disease. Table 3 shows clinical characteristics of these infants. In order to increase the power of the analysis, 9 control and 20 RSV-positive samples included in the microarray were also included in the qPCR.

**Table 3:** **Clinical characteristics of control and RSV-positive infants included in the qPCR analysis**

	**Control**		**Mild**		**Moderate**		**Severe**		**Sig.**
Number	19		16		7		19		
Age - months, median (IQR)	3	(1 – 5)	4	(1 – 7)	2	(1 – 4)	2	(1 – 4)	p = 0.6^1^
Male gender, n (%)	11	(58%)	7	(44%)	6	(86%)	9	(47%)	p = 0.5^2^
Weight - g, mean (SD)	5773	(1647)	7098	(2350)	6615	(1392)	5826	(1845)	p = 0.2^3^
Duration of symptoms - days, median (IQR)			4.5	(3 – 6)	5	(4 – 5)	4	(3 – 5)	p = 0.7^4^
Admission, n (%)			2	(13%)	7	(100%)	19	(100%)	
Length of stay, median (IQR)			0	(0 – 0)	3	(1 – 4)	4	(2 – 6)	p = 0.3^5^
Length of stay > 3 days, n (%)			0	(0%)	2	(29%)	10	(56%)	p = 0.2^6^
**Respiratory Distress**									
SpO2 -% on admission, mean (SD)			98	(2.5)	98	(2.5)	93	(6)	p = 0.003^7^
Respiratory rate /min on admission, mean (SD)			50	(14)	57	(8)	61	(13)	p = 0.06^7^
Respiratory rate score (max. 8), median (IQR)			1.5	(0 – 5)	4	(2 – 6)	4	(2 – 6)	p = 0.16^4^
Retraction score (max. 9), median (IQR)			1	(0 – 2)	5	(2 – 6)	2	(1 – 4)	p = 0.006^4^
Wheeze score (max. 8), median (IQR)			1	(0 – 3)	4	(4 – 5)	2	(2 – 4.5)	p = 0.053^4^
m-RDAI, median (IQR)			6	(1 – 8)	13	(9 – 16)	9	(7 – 13)	p = 0.004^4^
									p = 0.09^5^
**Treatments**									
Fluid supplement, n (%)							8	(42%)	
Intravenous fluids, n (%)							2	(11%)	
Nasogastric fluid, n (%)							7	(37%)	
Oxygen supplement, n (%)							16	(84%)	
CPAP, n (%)							3	(16%)	
Respirator, n (%)							0		

Note: There are no significant differences in age, weight or gender between control and RSV-positive groups. Duration of symptoms for disease subgroups was similar. The severe disease group had a lower SpO2 on admission, but tended to less respiratory distress than the moderate group, as measured by the m-RDAI. The retraction score in particular contributed to this difference. Length of hospital stay was statistically similar for moderate and severe disease groups, probably due to loss of power when excluding samples with poor RNA quality (when including all children regardless of RNA quality, the median length of stay and IQR are relatively unchanged, but Mann–Whitney p = 0.048, indicating a longer hospital stay for the severe disease group). The most common treatment for children with severe disease was oxygen supplementation.

^1^Mann-Whitney test, comparing control group with RSV-positive group.

^2^Fischer’s exact test, comparing control group with RSV-positive group.

^3^ *T*-test, comparing control group with RSV-positive group.

^4^Kruskal-Wallis test, comparing disease severity subgroups.

^5^Mann-Whitney test, comparing moderate and severe disease subgroups.

^6^Fischer’s exact test, comparing moderate and severe disease subgroups.

^7^One-way ANOVA, comparing disease severity subgroups.

**Figure 2:**
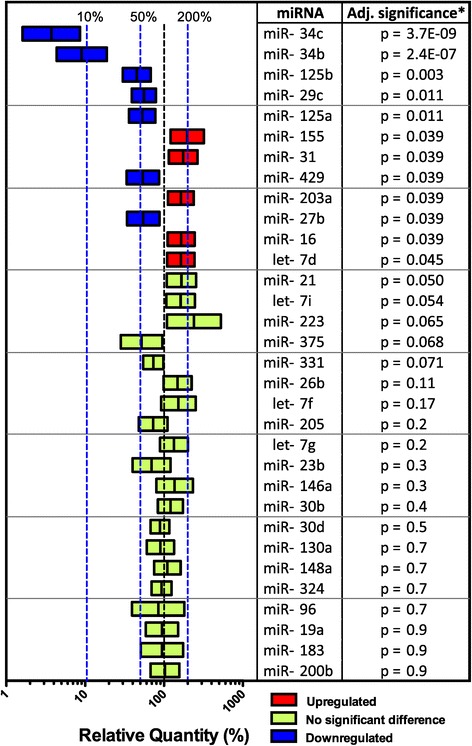
**Nasal mucosal miRNA expression in infants infected with** ***Respiratory syncytial virus,*** **compared to healthy controls.** Note: Samples from 42 children with RSV were compared to 19 control samples using qPCR for selected miRNA. Values presented are mean differences between disease and control groups with 95% confidence interval. *Multiple linear regression analysis with correction for RIN. p-values are adjusted for a false discovery rate of 5%.

Figure 2 shows the comparison of disease versus control for all 32 miRNAs analyzed by qPCR. miR-34b, miR-34c, miR-125b, miR-29c, mir125a, miR-429 and miR-27b were significantly downregulated. miR-155, miR-31, miR-203a, miR-16 and let-7d were significantly upregulated. Known immune functions of these miRNA are summarized in Table 4.

**Table 4:** **Functions, known targets and disease associations of differentially expressed miRNA**

**miRNA**	**Expression in this study**	**Functions in the immune system** ^**†**^	**Specific targets** ^**†**^	**Disease associations (Human studies)**
**Both innate and adaptive immune functions**				
**miR-29c**	Downregulation	Positive regulation of NFκB signaling [26]	TNFAIP3 [26]	Tuberculosis – upregulated [42]
		Influenza → Upregulation.^††^ Promotes apoptosis [43]	BCL2L2 [43]	Asthma – downregulated [37]
**miR-31**	Upregulation	Proliferation of myeloid cells [33]	E-selectin [44]	Influenza – downregulated [35]
**miR-155**	Upregulation	Proliferation of myeloid cells [33]	SHIP1 [33]	Influenza – downregulated [35]
		Dendritic cell maturation [34,45]	PU.1 [45], SOCS1, CD115, KPC1 [34]	
		Positive regulation of TLR signaling [18]	SOCS1 [18]	
		Required for dendritic, B and T-cell function [32]		
**let-7d**	Upregulation	Promotes T_h_1 polarization [40]	IL13 [40]	Asthma – downregulated [46]
		Endotoxin → Upregulation (TLR4 dep.) ^††^ [23]		
**Primarily innate immune functions**				
**miR-16**	Upregulation	Negative regulation of inflammation [47]	TNFα [47]	Sepsis – improved survival [48]
		Positive regulation of NFκB signaling [24]	SMRT [24]	
		Negative regulation of NFκB signaling [28]	IKKα [28]	
		Endotoxin → Upregulation (TLR4 dep) ^††^ [23]		
**miR-27b**	Downregulation	TLR4/NFκB induced nitric oxide production [49]	KSRP [49]	Asthma – downregulated [37]
		Endotoxin → Upregulation (NFκB dep) ^††^ [50]		
**miR-34b**	Downregulation	Endotoxin → Upregulation (TLR4 dep) ^††^ [23]		Asthma – downregulated [37]
**miR-34c**	Downregulation	Negative regulation of NFκB signaling; DAMPs* → Upregulation [16]	IKKγ [16]	Asthma – downregulated [37]
		In cord blood monocytes: IFNγ → Upregulation^††^; Endotoxin → no regulation^††^ [17]		
**miR-125a**	Downregulation	Positive regulation of NFκB signaling [19]	TNFAIP3 [19]	
		Inhibits CCL5 production [30]	KLF13 [30]	
		Macrophage polarization [27,51]		
**miR-125b**	Downregulation	Positive regulation of NFκB signaling [19]	TNFAIP3 [19]	CERS** – upregulated [52]
		Negative regulation of NFκB signaling [18]	MyD88 [18]	
		Activates Macrophages [20]	IRF4 [20]	
		Modulates dendritic cell differentiation [21]	PRDM1 [21]	
		Maintains naïve state in CD4+ T-cells [53]	IFNγ, IL2RB, IL10RA, PRDM1 [53]	
		Negative regulation of inflammation [22]	TNFα [22]	
**miR-203a**	Upregulation	Negative regulation of NFκB signaling [25]	Myd88 [25]	Asthma – upregulated [38]
		Negative regulation of inflammation [54]	TNFα, IL24 [54]	
**No known immune functions**				
**miR-429**	Downregulation			

†: The functions described are extracted from a literature search April 2014, excluding studies of cancer. This table is not an exhaustive description of all known functions for each miRNA. All studies were carried out in vitro in specific cell lines, and caution should be taken when extrapolating the data to other cells or biological systems, including clinical disease. Specific miRNA functions and mRNA-target verification have in most cases been determined both by removing the miRNA from the biological system and by stimulating the system with the miRNA. miRNA upregulation after cell stimulation has usually been verified by simple qPCR. miRNA responses to cell stimulation have been included in the table if deemed relevant to virus infection or TLR/NFκB signaling.

††: X → Upregulation (Y dep): indicates that in-vitro stimulation of a cell line with molecule or pathogen X causes upregulation of the miRNA, and that this is dependent upon protein Y.

*DAMPs: Damage-associated molecular pattern molecules.

**CERS - Chronic Eosinophilic Rhinosinusitis.

### Disease subgroups

**Figure 3:**
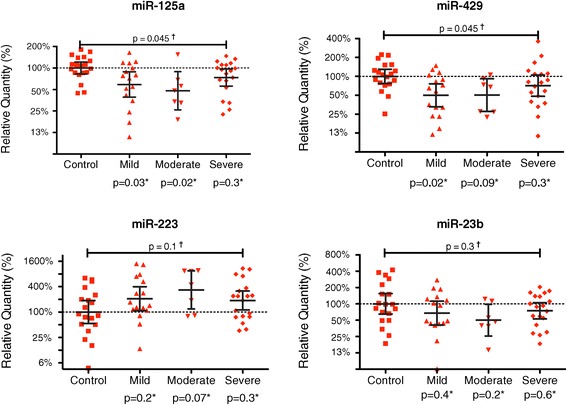
**Nasal mucosal miRNA expression according to disease severity in infants infected with** ***Respiratory syncytial virus*** **.** Note: Relative quantities of 4 miRNAs assessed for differential expression between disease severity subgroups. Results are calibrated to the mean of the control group, indicated by the dashed line at a relative quantity 100%. Plotted lines indicate the mean with 95% confidence interval for each group. Numbers in each group: control 19; mild 16; moderate 7; severe 19. For miR-125a there is downregulation in mild and moderate disease groups, but not in the severe group. For miR-429 there is a similar pattern, with downregulation in the mild group but not the severe group. ^**†**^ One-way ANOVA, adjusted for a false discovery rate of 5%. * Dunnett’s post-hoc *t*-test after One-way ANOVA, in which mild, moderate and severe disease severity subgroups are compared individually to the control group, with correction for multiple testing. Up- or downregulation is therefore as compared to the control group.

miR-125a and mir-429 showed evidence of differential expression between disease subgroups (Figure 3). Compared to control, the mild and moderate disease subgroups show miR-125a downregulation, but not the severe disease subgroup. miR-429 shows a similar pattern, except that the downregulation in the moderate group is not quite significant.

## Discussion

We have profiled miRNA expression in nasal epithelial cells of infants with acute RSV disease, compared to healthy controls. In addition we find differential expression between mild, moderate and severe disease groups for miR-125a and miR-429.

Bronchiolitis is a disease in which the entire respiratory tract is affected, with both upper and lower respiratory symptoms in the majority of children. Ethical considerations limited this investigation to the nasal mucosa since we were interested in infants not receiving intensive care treatment. Several cell types were present in our samples: granulocytes, squamous epithelial cells and ciliary epithelial cells. We are therefore unable to make direct conclusions about miRNA expression in cells of the lower respiratory tract or individual cell types. We do, however, consider that the nasal mucosa is a reasonable site to examine the immunological response to RSV infection. The nasal cavity is the primary site of RSV infection, and children typically have coryza for several days prior to lower respiratory symptoms. Differences in the nasal mucosal immune response may increase the risk of severe disease, for example by promoting dissemination of viral particles to the lower respiratory tract, or by increasing mucous production and inflammation.

RSV preferentially infects epithelial cells of the respiratory mucosa. We differentiated between disease severities in the setting of patients routinely admitted to hospital, such that severe disease patients were largely treated on the pediatric ward, not the intensive care unit. Severe disease was distinguished from moderate disease on the basis of supplemental oxygen or fluids, or use of CPAP. It is therefore interesting that on admission, those in the moderate disease group tended to more respiratory distress than those in the severe disease group, as measured by the m-RDAI (Table 3). This is a similar pattern to the PCR findings for miR-125a, and miR-429, where there is regulation in mild or moderate disease groups that is not present in the severe disease group. Increased signs of respiratory effort may be a protective factor, and infants who do not show these signs may either be too tired to increase their work of breathing, or may not have this capacity for other reasons - for example the body’s drive (immunological or otherwise) to respond to infection. Clinical findings should, however, be interpreted with caution because the m-RDAI was only measured on admission. If we had re-assessed respiratory effort during the course of the admission we may have discovered an increased score prior to oxygen or fluid administration. In this prospective study, an arbitrary m-RDAI cut-off was used to distinguish mild from moderate disease groups in admitted patients. Only two mild disease patients included in the final analysis were admitted, and both of these had an m-RDAI score of 6, largely due to tachypnea, with little wheeze and few retractions. We therefore consider these patients to be correctly categorized.

26 miRNAs were differentially regulated in the microarray. 11 of these were also differentially regulated in the qPCR. Of the 6 miRNA not differentially regulated in the microarray, one (miR-155) was differentially regulated in the qPCR. For miRNA regulated in both microarray and qPCR, the pattern of regulation was similar in the two analyses when grouping all RSV positive children together (Figures 1 and 2). On subgroup analysis, miRNA group C (see Figure 1) is of interest. Group C includes miRNA downregulated in mild disease but not in severe disease. miR-125a and miR-429 are in this group, and qPCR subgroup analysis was consistent with the microarray. miR-125b, miR-27b and miR-29c were also in this group but did not show differences between disease groups. The other 12 group C miRNA were not differentially regulated in the qPCR. The use of clinical samples with a high degree of biologically variation, the fact that microarray is less precise than qPCR, the low number of samples in each group, and the increased number of samples in the qPCR may explain differences between microarray and qPCR results.

The majority of miRNAs regulated in this study have documented functions within the immune system (see Table 4). Expression levels of miR-34c, miR-34b and miR-125b were reduced more than two-fold compared to control, with significance levels several orders of magnitude greater than the other regulated miRNAs, suggesting that downregulation of these miRNAs in particular may be important in the immune response to RSV. One report described miR-34c upregulation by adult peripheral blood monocytes on exposure to damage-associated molecular pattern molecules. In the same paper miR-34c negatively regulated NF-κB signaling by inhibition of Inhibitor of IKKγ [16]. Another study showed miR-34c upregulation in umbilical cord blood monocytes on exposure to the Th1 cytokine interferon-γ, but not on exposure to lipopolysaccharide [17]. miR-125b has been well studied, and a number of functions within the immune system are described, including regulation of NF-κB signaling [18,19], macrophage activation [20], modulation of dendritic cell differentiation [21] and direct targeting of TNFα [22]. miR-34b has been less studied in immunity, but was upregulated in mouse lung tissue after injection of lipopolysaccharide [23].

NF-κB signaling is modulated by 7 miRNAs regulated in our study: miR-16, miR-29c, miR-34c, miR-125a, miR-125b, miR-155 and miR-203a [16,18,19,24-28]. NF-κB activation following RSV-antigen binding to the pathogen recognition receptors TLR 4 or RIG-1 is a primary stage in the immunological response to RSV [29]. However, excessive NF-κB activation may also have deleterious effects, and negative regulation is also important. The miRNA regulation pattern in this study implies both positive and negative regulation of NF-κB, consistent with fine-tuning of the immune response to RSV in our samples.

Factors that might cause some infants to have severe disease whilst most infants have mild disease are poorly understood and may aid development of preventative measures and treatments for RSV. We therefore aimed to identify differences in miRNA expression between mild and severe disease. The variation in expression of miR-125a and miR-429 according to disease severity is particularly interesting in the context of innate immunity. miR-125a is a positive regulator of NF-κB via negative regulation of the inhibitory protein TNFAIP3 [27], and a negative regulator of CCL5, an important cytokine in both innate and adaptive immune systems [30]. miR-125a is overexpressed in human macrophages, in an activated phenotype produced by NF-κB activation [27]. Downregulation of miR-125a in children with mild and moderate disease may thus represent reduced macrophage activation and inhibited NF-κB signaling, which may protect against an excessive innate immune response with a more severe disease phenotype. miR-429 can reactivate dormant Epstein-Barr virus in B-cells [31]. To our knowledge, other functions of miR-429 within the immune system or in RSV disease have not been described.

Upregulation of miR-155 is consistent with an acute inflammatory response. This miRNA is expressed in dendritic cells and in T and B- lymphocytes and is necessary for their function [32]. miR-155 is produced on TLR4 mediated NF-κB activation and positively regulates myeloid proliferation [33] and dendritic cell maturation [34], which is a necessary step for dendritic cell migration to lymph nodes and antigen presentation.

Several studies have examined in vitro responses to RSV. RSV-stimulation upregulates miR-375 and let-7i in human bronchial epithelial cells [8,9], and let-7f in a human alveolar cell line [7]. These miRNA were differentially regulated in our microarray, but not in the qPCR (FDR-adjusted p = 0.054, 0.068 and 0.17 for let-7i, miR-375 and let-7f, respectively – see Figure 2). A number of other miRNA identified in these studies were not regulated in our microarray. It is probable that differences in findings between our study and these in vitro studies are due to the cell type analyzed. Our cytology specimens included both nasal respiratory epithelial cells and granulocytes, whilst the in-vitro studies included alveolar or bronchial epithelial cell lines. In addition, the increased biological variation inherent to our clinical samples is likely to reduce the power of our study to observe differences in expression.

To our knowledge, miRNAs have not been studied in clinical disease with respiratory viruses other than in blood samples of patients with influenza A [35,36]. This is the first investigation of miRNA expression in clinical samples from RSV-positive infants. Two clinical studies profiled miRNA expression in bronchial epithelial brushings from asthmatic adults [37,38]. Findings included upregulation of miR-203a and downregulation of miR-27b, miR-29c, miR-34b and miR-34c – an identical expression pattern to the infants in our study. Early-life RSV infection is associated with later development of asthma [39], making this finding particularly interesting. However, we also found downregulation of let-7d, which targets asthma-associated genes in vitro [40]. Further studies of the role of these miRNA in RSV disease may provide information on mechanisms underlying the association between infantile RSV disease and later development of asthma.

## Conclusions

In summary, we have profiled miRNA expression patterns in the nasal epithelium of infants with acute RSV disease. We find an expression pattern consistent with modulation of both innate and adaptive immune systems, including a number of miRNAs involved in NF-κB regulation or associated with asthma. miR-125a and miR-429 were regulated in mild but not severe disease, warranting further investigation of their role as a regulator of the immune response to RSV.
